# Development of early prediction model for pregnancy-associated hypertension with graph-based semi-supervised learning

**DOI:** 10.1038/s41598-022-15391-4

**Published:** 2022-09-22

**Authors:** Seung Mi Lee, Yonghyun Nam, Eun Saem Choi, Young Mi Jung, Vivek Sriram, Jacob S. Leiby, Ja Nam Koo, Ig Hwan Oh, Byoung Jae Kim, Sun Min Kim, Sang Youn Kim, Gyoung Min Kim, Sae Kyung Joo, Sue Shin, Errol R. Norwitz, Chan-Wook Park, Jong Kwan Jun, Won Kim, Dokyoon Kim, Joong Shin Park

**Affiliations:** 1 Department of Obstetrics and Gynecology, Seoul National University College of Medicine, 101 Daehak-ro, Jongno-gu, Seoul, 03080 South Korea; 2 Department of Biostatistics, Epidemiology and Informatics, The Perelman School of Medicine, University of Pennsylvania, B304 Richards Building, 3700 Hamilton Walk, Philadelphia, PA 19104-6116 USA; 3 Department of Obstetrics and Gynecology, Seoul National University Hospital, Seoul, South Korea; 4 Seoul Women’s Hospital, Incheon, South Korea; 5 Department of Obstetrics and Gynecology, Seoul Metropolitan Government, Seoul National University Boramae Medical Center, Seoul, South Korea; 6 Department of Radiology, Seoul National University College of Medicine, Seoul, South Korea; 7 Department of Radiology, Yonsei University College of Medicine, Seoul, South Korea; 8 Department of Internal Medicine, Seoul National University College of Medicine, Seoul, South Korea; 9 Department of Internal Medicine, Seoul Metropolitan Government, Seoul National University Boramae Medical Center, Seoul, South Korea; 10 Department of Laboratory Medicine, Seoul National University College of Medicine, Seoul, South Korea; 11 Department of Laboratory Medicine, Seoul Metropolitan Government, Seoul National University Boramae Medical Center, Seoul, South Korea; 12 Department of Obstetrics and Gynecology, Tufts University School of Medicine, Boston, MA USA; 13 Institute for Biomedical Informatics, University of Pennsylvania, Philadelphia, PA USA; 14 Department of Obstetrics and Gynecology, Korea University College of Medicine, Seoul, South Korea

**Keywords:** Computational biology and bioinformatics, Risk factors

## Abstract

Clinical guidelines recommend several risk factors to identify women in early pregnancy at high risk of developing pregnancy-associated hypertension. However, these variables result in low predictive accuracy. Here, we developed a prediction model for pregnancy-associated hypertension using graph-based semi-supervised learning. This is a secondary analysis of a prospective study of healthy pregnant women. To develop the prediction model, we compared the prediction performances across five machine learning methods (semi-supervised learning with both labeled and unlabeled data, semi-supervised learning with labeled data only, logistic regression, support vector machine, and random forest) using three different variable sets: [a] variables from clinical guidelines, [b] selected important variables from the feature selection, and [c] all routine variables. Additionally, the proposed prediction model was compared with placental growth factor, a predictive biomarker for pregnancy-associated hypertension. The study population consisted of 1404 women, including 1347 women with complete follow-up (labeled data) and 57 women with incomplete follow-up (unlabeled data). Among the 1347 with complete follow-up, 2.4% (33/1347) developed pregnancy-associated HTN. Graph-based semi-supervised learning using top 11 variables achieved the best average prediction performance (mean area under the curve (AUC) of 0.89 in training set and 0.81 in test set), with higher sensitivity (72.7% vs 45.5% in test set) and similar specificity (80.0% vs 80.5% in test set) compared to risk factors from clinical guidelines. In addition, our proposed model with graph-based SSL had a higher performance than that of placental growth factor for total study population (AUC, 0.71 vs. 0.80, p < 0.001). In conclusion, we could accurately predict the development pregnancy-associated hypertension in early pregnancy through the use of routine clinical variables with the help of graph-based SSL.

## Introduction

Pregnancy-associated hypertension (HTN) is one of the most serious complications of pregnancy, affecting 1–8% of pregnancies worldwide^1–3^. It results in increased mortality and morbidity for both pregnant women and neonates^3,4^. Recently, prophylactic low-dose aspirin has been reported to reduce the incidence of pregnancy-associated HTN in high-risk pregnancies^5^. Moreover, starting aspirin early in pregnancy appears to be more effective than starting in late pregnancy^6–8^.

Clinical guidelines recommend several risk factors—including chronic hypertension, diabetes, and/or pregnancy-associated HTN in a prior pregnancy—to identify pregnancies at high-risk to guide aspirin prophylaxis; however, these factors have low predictive accuracy^6,9^. Other algorithms have been developed to predict pregnancy-associated HTN using biomarkers such as placental growth factor (PlGF) and/or ultrasonographic uterine artery Doppler velocimetry, but this strategy can be challenging to introduce in routine clinical practice^10^. Indeed, the measurement of uterine artery Doppler velocimetry and PIGF in early pregnancy may not be possible in all pregnant women, especially in low-resource areas. Moreover, measurement of uterine artery Doppler or PlGF during early pregnancy in low-risk pregnant women has not been established in routine practice because of high cost for measurement of PlGF and ultrasound examination.

Accurate prediction for pregnancy-associated HTN in early pregnancy remains an unmet need for clinicians. Machine learning approaches have recently been widely adopted in clinical research for the development of predictive models for complex diseases. However, only a few studies have attempted to use machine learning to predict the risk of pregnancy-associated HTN. Furthermore, these studies developed prediction models using only clinical variables retrieved in late pregnancy^11,12^ or have failed to show robust performance in early pregnancy^13^.

Prediction using supervised learning (traditional machine learning) suffers from the presence of unlabeled data (patients lost to follow up and whose outcomes are unknown), which is inevitable in real-world data. Semi-supervised learning (SSL), which is capable of using both labeled data (from patients whose outcomes are known) and unlabeled data to infer relations across patients, can be used to increase the amount of information available in the dataset, resulting in better performance. Several studies have been conducted to incorporate both labeled and unlabeled data using SSL for a predictive model for cancer survivability such as breast and ovarian cancer^14–16^. Furthermore, the early identification of women at high-risk for pregnancy-associated HTN will allow for patient stratification, classifying high-risk patients who may share a common underlying disease pathophysiology, and thus may be characterized by several common features. Considering these two points, we adopted graph-based semi-supervised learning (SSL) for the prediction of pregnancy-associated HTN in early pregnancy. Graph-based SSL can perform effective prediction through the propagation of label information according to the structure of patient-derived networks^17,18^.

To this end, the purpose of the current study was to develop a prediction model for pregnancy-associated HTN in first trimester using routine clinical variables. We selected important clinical variables using various feature selection methods, and optimized and validated the prediction model using graph-based SSL.

## Results

### Subject population

During the study period, a total of 1742 women were enrolled until 14 weeks of gestation. Women with previable preterm birth or abortion (n = 11), withdrawal of consents (n = 30), or who received aspirin prophylaxis (n = 3) were excluded, leaving 1,698 women. Exclusion of patients with missing variables yielded a final sample of 1404 women for the final analysis of graph-based SSL with both labeled and unlabeled data (${\mathrm{SSL}}_{\mathrm{L}+\mathrm{U}}$). Among these, 1347 women that were followed up until delivery (labeled data) were included in the other machine learning analyses (${\mathrm{SSL}}_{\mathrm{L}}$, LR, SVM, and RF). Our unlabeled data of patients included 57 women with incomplete follow-up. Supplementary Table 1 presents women who completed follow-up and those who were lost to follow-up. There were no significant differences in clinical variables between the two groups.

In the 1347 women that completed follow-up (labeled data), 2.4% (33/1347) developed pregnancy-associated HTN. Table 1 shows the clinical characteristics, conventional risk factors recommended by ACOG, and pregnancy outcomes of the study population. Among women who developed pregnancy-associated HTN, only 42.4% were classified as high risk according to the ACOG guideline. As regards pregnancy outcomes, patients who developed pregnancy-associated HTN delivered in earlier gestational age than those who did not. Neonates who were born to mothers with pregnancy-associated HTN had lower birthweight and higher risk of neonatal intensive care unit admission than those who were not (Table 1).

**Table 1:** Baseline clinical features and pregnancy outcomes of the study population.

Characteristics	Pregnancy-associated HTN (−) (n = 1314)	Pregnancy-associated HTN ( +) (n = 33)	*P-*value
**Baseline characteristics**			
Maternal age (years)	32.3 ± 4.0	33.0 ± 4.7	0.518
Nulliparity	671 (51.1%)	21 (63.6%)	0.211
**Risk factors in clinical guidelines**			
** (1) High risk by the presence of either high- or moderate-risk factors**	**188 (14.3%)**	**14 (42.4%)**	** < 0.001**
** (2) High risk by the presence of one or more high-risk factors**	**27 (2.1%)**	**9 (27.3%)**	** < 0.001**
a. Previous history of preeclampsia	8 (0.6%)	4 (12.1%)	< 0.001
b. Chronic hypertension	7 (0.5%)	4 (12.1%)	< 0.001
c. Pregestational diabetes	15 (1.1%)	2 (6.1%)	0.063
d. Renal disease	2 (0.2%)	0 (0.0%)	1.000
e. Autoimmune disease	1 (0.1%)	0 (0.0%)	1.000
** (3) High risk by the presence of two or more moderate-risk factors**	**166 (12.6%)**	**9 (27.3%)**	**0.030**
a. First pregnancy	671 (51.1%)	21 (63.6%)	0.211
b. Old age (≥ 35 year)	385 (29.3%)	12 (36.4%)	0.493
c. Obesity (BMI > 30 kg/m^2^)	70 (5.3%)	6 (18.2%)	0.003
d. African race	0 (0%)	0 (0%)	(−)
**Pregnancy outcome**			
Gestational age at delivery (weeks)	39.0 ± 1.3	36.3 ± 3.0	< 0.001
Gestational diabetes	72 (5.7%)	6 (20.0%)	0.007
Birthweight at delivery (kg)	3.2 ± 0.4	2.6 ± 0.8	< 0.001
Infant sex (male)	675 (51.4%)	17 (51.5%)	1.000
Infant admission to NICU	53 (4.0%)	9 (27.3%)	< 0.001

Data are presented as proportion (%) or mean standard ± deviation.

*BMI* body mass index, *HTN* hypertension, *NICU* neonatal intensive care unit.

The study population was divided into training and test sets based upon the enrollment year of pregnant women (training set—971 patients enrolled between 2014 and 2017, including 22 women who developed pregnancy-associated HTN; test set—376 patients enrolled between 2018 and 2019, including 11 women who developed pregnancy-associated HTN). The prediction model for pregnancy-associated HTN was developed using the training set, and evaluated in the test set.

### Variables ranking and selection

Among 32 routine clinical variables, 11 important variables were selected by backward elimination during training according to integrated rankings. The selected variables were as follows, listed in order of their importance (Table 2): diastolic BP in early pregnancy, systolic BP in early pregnancy, diastolic BP in late first trimester, hemoglobin level measured in the first trimester, systolic BP in late first trimester, maternal BMI before pregnancy, maternal age, maternal BMI in late first trimester, history of preeclampsia in previous pregnancy, maternal weight in late first trimester, and maternal weight before pregnancy. Among variables, laboratory or physical measures were evaluated at 7.7 ± 1.2 weeks for early first trimester and at 12.4 ± 0.5 weeks for late first trimester. Table 3 compares the top 11 important variables according to the development of pregnancy-associated HTN. Selected 11 variables were different between the two groups.

**Table 2:** Rank of top 11 important variables selected from various machine learning methods; support vector machine with recursive feature elimination (SVM-RFE), logistic regression with recursive feature elimination (LR-RFE), random forest using gini index (RF-gini), and random forest using information entropy (RF-entropy).

Clinical variables	Ranks by selection methods				Integrated ranking^a^
	SVM (RFE)	LR (RFE)	RF (gini)	RF (entropy)	
Diastolic blood pressure (BP) in early pregnancy	1	1	1	1	1.00
Systolic BP in early pregnancy	1	2	2	2	1.68
Diastolic BP in late first trimester	3	5	4	4	3.94
Hemoglobin level measured in the first trimester	4	14	5	5	6.17
Systolic BP in late first trimester	16	17	3	2	6.36
BMI before pregnancy	7	3	10	10	6.77
Maternal age	5	4	14	14	7.91
BMI in late first trimester	10	9	9	6	8.35
History of preeclampsia in previous pregnancy	12	6	6	12	8.49
Weight in late first trimester	8	7	12	11	9.27
Weight before pregnancy	6	10	11	13	9.62

Early pregnancy, measured at 7.7 ± 1.2 weeks; late first trimester, measured at 12.4 ± 0.5 weeks.

*BMI* body mass index, *BP* blood pressure.

^a^To combine/aggregate four different rankings, we apply the geometric mean which is defined as ${\left({\prod }_{i-1}^{n}{r}_{i}\right)}^\frac{1}{n}=\sqrt[n]{{r}_{1}{r}_{2}\dots {r}_{n}}$ where ${r}_{i}$ is the variable ranks in $i$th selection methods.

**Table 3:** Analysis of selected top 11 important variables in the study population.

(a) In training set			
Selected variables	Pregnancy-associated HTN (−) (n = 949)	Pregnancy-associated HTN ( +) (n = 22)	*P-*value
**Variables before pregnancy**			
Maternal age	32.2 ± 3.9	33.3 ± 4.9	0.383
History of preeclampsia	7 (0.7%)	2 (9.1%)	0.016
Weight before pregnancy	57.9 ± 10.1	69.0 ± 13.9	< 0.001
BMI before pregnancy	22.2 ± 3.6	26.2 ± 5.2	< 0.001
**Variables in the first trimester**			
Systolic BP in early pregnancy	113.3 ± 11.4	132.7 ± 17.2	< 0.001
Diastolic BP in early pregnancy	67.3 ± 8.4	80.5 ± 10.8	< 0.001
Systolic BP in late first trimester	112.6 ± 11.4	126.3 ± 14.4	< 0.001
Diastolic BP in late first trimester	67.6 ± 8.6	77.9 ± 9.8	< 0.001
Weight in late first trimester	58.8 ± 10.1	69.7 ± 14.0	< 0.001
BMI in late first trimester	22.5 ± 3.6	26.4 ± 5.2	< 0.001
Hemoglobin level in the first trimester	12.6 ± 1.0	13.6 ± 1.0	< 0.001

(b) In test set			
Selected variables	Pregnancy-associated HTN (−) (n = 365)	Pregnancy-associated HTN ( +) (n = 11)	*P-*value
**Variables before pregnancy**			
Maternal age	32.4 ± 4.2	32.4 ± 4.3	0.891
History of preeclampsia	1 (0.3%)	2 (18.2%)	0.002
Weight before pregnancy	58.5 ± 10.9	65.1 ± 16.2	0.124
BMI before pregnancy	22.3 ± 3.8	25.5 ± 6.2	0.062
**Variables in the first trimester**			
Systolic BP in early pregnancy	115.4 ± 11.1	130.5 ± 11.4	< 0.001
Diastolic BP in early pregnancy	68.8 ± 8.4	79.1 ± 10.2	0.001
Systolic BP in late first trimester	114.4 ± 11.4	126.4 ± 10.1	0.001
Diastolic BP in late first trimester	68.5 ± 8.2	77.0 ± 11.8	0.011
Weight in late first trimester	59.3 ± 10.7	65.2 ± 16.5	0.169
BMI in late first trimester	22.6 ± 3.7	25.5 ± 6.1	0.089
Hemoglobin level in the first trimester	12.7 ± 1.0	13.2 ± 1.0	0.319

Early pregnancy, measured at 7.7 ± 1.2 weeks; late first trimester, measured at 12.4 ± 0.5 weeks.

*BMI* body mass index, *BP* blood pressure.

### Development of prediction model for pregnancy-associated hypertension

Table 4 shows the overall performance comparison for each model with respect to sets of [a], [b], and [c] in training set, measured in terms of AUROC, sensitivity, specificity, positive predicted value (PPV), and negative predicted value (NPV). Overall, ${\mathrm{SSL}}_{\mathrm{L}+\mathrm{U}}^{[\mathrm{b}]}$ (SSL using the top 11 important variables) achieved an average AUROC of 0.885 which was the best of the five models and was selected as the proposed prediction model. The p-values of pairwise t-tests between proposed model (${\mathrm{SSL}}_{\mathrm{L}+\mathrm{U}}^{[\mathrm{b}]}$) and other representative models (${\mathrm{SSL}}_{\mathrm{L}}^{[\mathrm{b}]}$, LR^[b]^, SVM^[b]^, RF^[b]^) were all < 0.001, respectively. Moreover, in terms of performance comparison for 3 different sets of each model ([a], [b], and [c]), the AUROC for all of the models using [b] was better than that of the others. The AUROC of the proposed model (${\mathrm{SSL}}_{\mathrm{L}+\mathrm{U}}^{[\mathrm{b}]}$) using 11 selected variables was increased by 24.5% ($=\left(\frac{0.885-0.710}{0.710}\right)\times 100\mathrm{\%}$) on average, compared to model (${\mathrm{SSL}}_{\mathrm{L}+\mathrm{U}}^{[\mathrm{a}]}$) using variables from clinical guidelines, suggesting that the selected variables were more effective than conventional risk factors recommended by ACOG guidelines.

**Table 4:** Performance comparison in test set.

Models	AUROC	Sensitivity	Specificity	PPV	NPV
${\mathrm{SSL}}_{\mathrm{L}+\mathrm{U}}^{[\mathrm{b}]}$	**0.811**	**0.727**	**0.800**	**0.099**	**0.989**
${\mathrm{LR}}^{[\mathrm{b}]}$	0.762	0.636	0.663	0.054	0.984
${\mathrm{SVM}}^{[\mathrm{b}]}$	0.701	0.719	0.795	0.096	0.989
${\mathrm{RF}}^{[\mathrm{b}]}$	0.725	0.725	0.666	0.062	0.987
Risk factors	–	0.455	0.805	0.066	0.980

Risk factors: conventional risk factors recommended by American College of Obstetricians and Gynecologists.

*AUROC* area under the ROC curve, *PPV* positive predicted value, *NPV* negative predicted value; [a]: models with variables from clinical guidelines, [b] models with selected important variables, and [c] models with all routine variables.

The performances of the best model are in bold.

**Figure 1:**
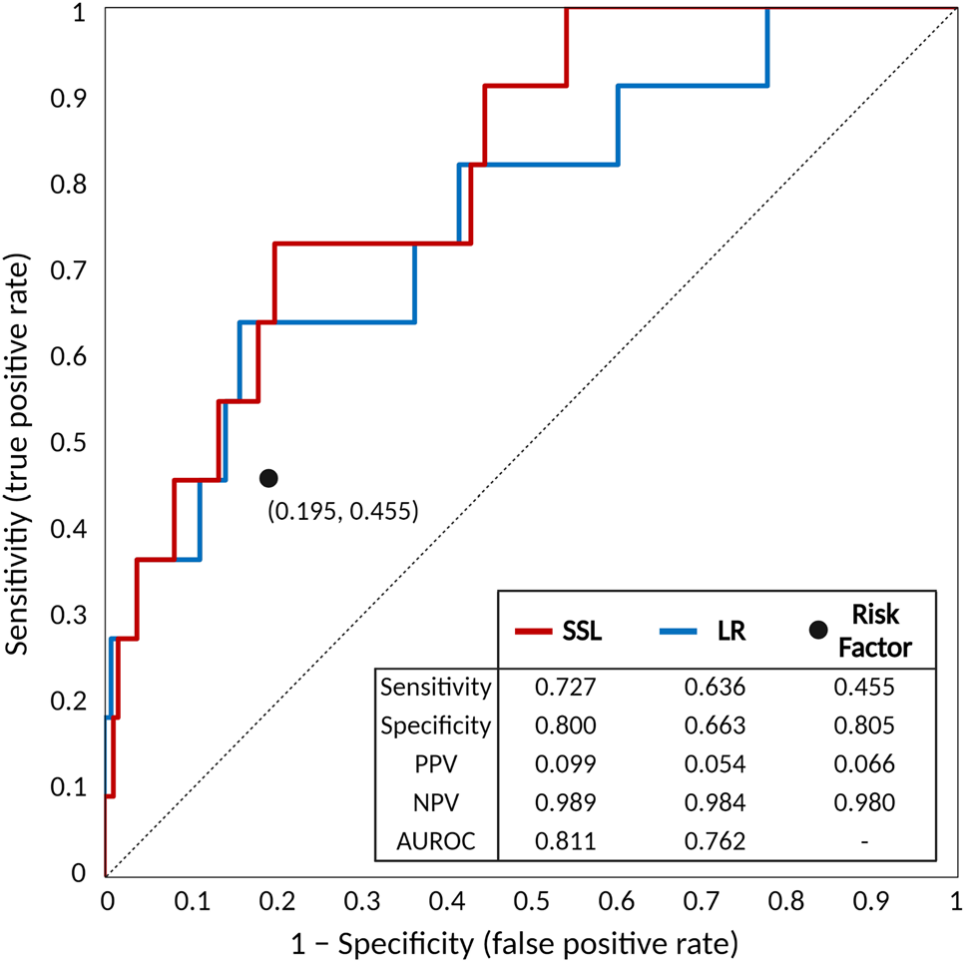
Receiver operating characteristic curve of proposed prediction model with graph-based semi-supervised learning in test set population (enrolled 2018–2019). Risk factors: conventional risk factors recommended by American College of Obstetricians and Gynecologists.

The performance of the proposed prediction model (${\mathrm{SSL}}_{\mathrm{L}+\mathrm{U}}^{[\mathrm{b}]}$) (the graph-based SSL using top 11 important variables) was also evaluated and validated in the test set, with an AUROC of 0.811. Figure 1 shows the performance comparison between the proposed prediction model and the logistic regression model. The graph-based SSL had higher AUROC than that of logistic regression model (0.811 vs. 0.762). In addition, the proposed prediction model showed higher sensitivity (72.7% vs 45.5%) and similar specificity (80.0% vs 80.5%) compared to risk factors from clinical guidelines, in test set.

### Comparing proposed models vs. PlGF

**Figure 2:**
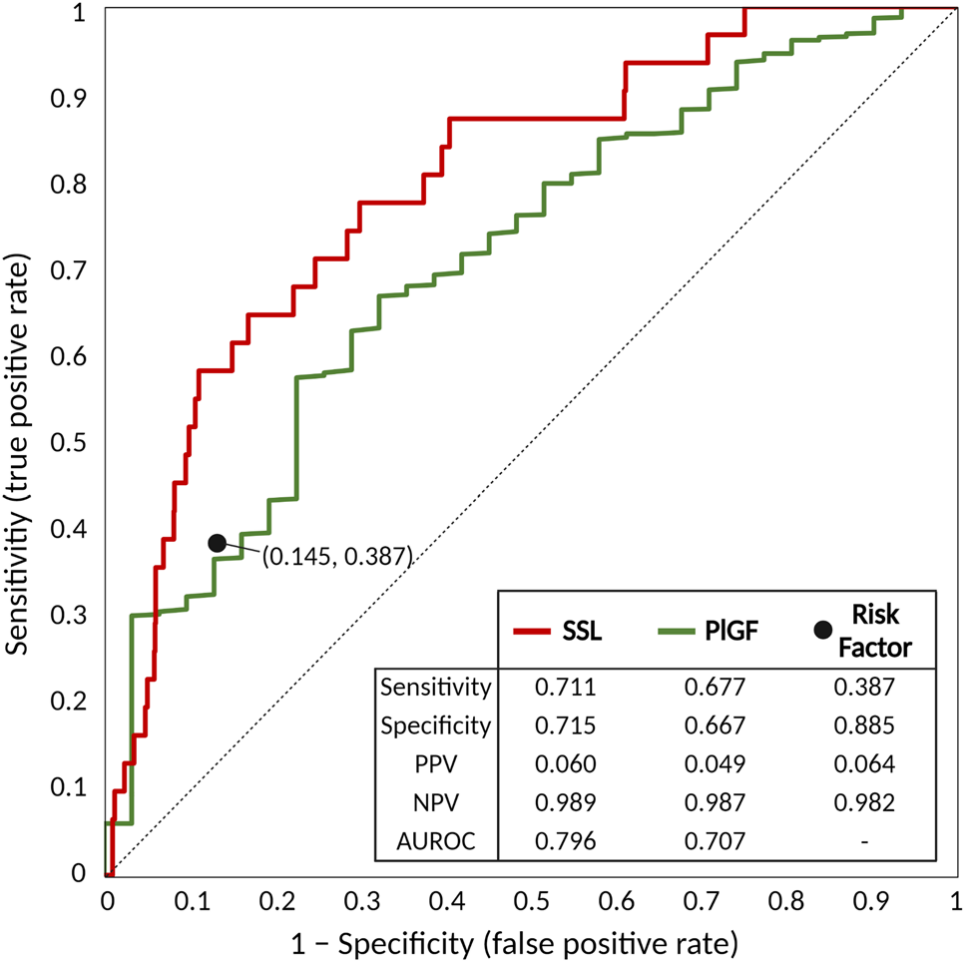
Receiver operating characteristic curve of proposed prediction model vs. placental growth factor (PlGF). Risk factors: conventional risk factors recommended by American College of Obstetricians and Gynecologists. *AUROC* the area under the ROC curve, *NPV* negative predicted value, *PlGF* placental growth factor, *PPV* positive predicted value, *SSL* semi-supervised learning.

In the study population of 1347 women with known outcomes, maternal blood was collected in the first trimester that was available to measure PlGF, a well-known biomarker for early prediction of pregnancy-associated HTN. To compare the prediction performance between graph-based SSL and PlGF, we performed an external experiment; the entire population was divided into patients with or without PlGF and set as a training set (labeled set) and a test set (unlabeled set), respectively. For this experiment, the prediction model was constructed with graph-based SSL from patients who did not have PlGF measurements (92 patients whose blood sample was not available for measurement, including two patients who developed pregnancy-associated HTN and 90 patients who did not) and was validated in the 1255 patients that did have PlGF measurements. This experiment demonstrates one of the advantages of the semi-supervised approach, as it is possible to predict outcomes with a small amount of labeled data using SSL. Figure 2 compares the ROC curve of the proposed prediction model with that of PlGF and conventional clinical guidelines. The conventional clinical guideline showed sensitivity of 42.4% (14/33) and specificity of 85.7% (1,126/1,314), and PlGF had lower AUROC than the prediction model from graph-based SSL (0.71 vs. 0.80, p < 0.001 by Delong test).

### A network of patients from graph-based SSL

**Figure 3:**
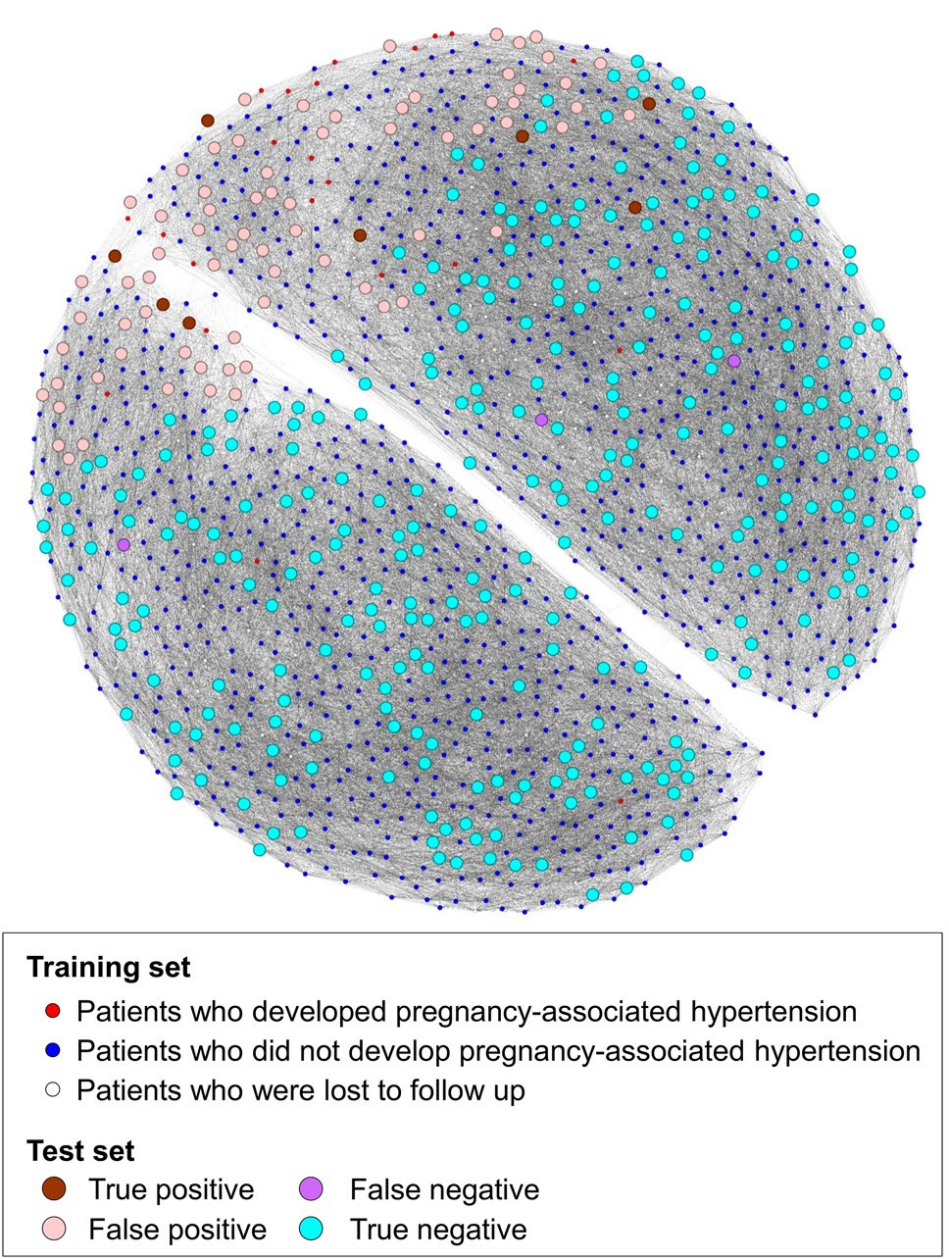
The patient-derived network with 1404 pregnant women. Training set (enrolled in 2014–2017); Test set (enrolled in 2018–2019).

Figure 3 shows the constructed patients’ network in the test set, derived from the proposed prediction model with graph-based SSL. Red dots represent those who develop pregnancy-associated HTN and grey dots represent patients who did not develop pregnancy-associated HTN. Red dots are proximally close to one another, suggesting that patients who developed pregnancy-associated HTN showed similarity between patients, which enabled positive prediction for pregnancy-associated HTN. White dots represent patients who were lost to follow-up, and these patients were also used in model development from their characteristics and contributed to the increased model performance of graph-based SSL.

## Discussion

### The major findings of this study

(1) With aggregating ranks, the top 11 variables were selected according to their importance of describing pregnancy-associated HTN; (2) The graph-based SSL using the selected 11 important variables achieved the best average prediction performance, with higher sensitivity and similar specificity compared to risk factors from clinical guidelines; (3) In addition, the proposed model with graph-based SSL had higher AUROC than that of PlGF.

### Results and clinical implications

Existing clinical guidelines recommend several risk factors to determine which pregnant women are at high-risk of developing pregnancy-associated HTN and therefore candidates for aspirin prophylaxis in early pregnancy^19–24^. However, recent studies using these guidelines reported low predictive performance. For example, in a 2017 European study, the British guideline (National Institute for Health and Care Excellence, NICE) showed a detection rate of around 40%with false-positive rate of 10%^25^, and in a 2019 Asian study, the NICE guideline showed a detection rate of 26% with 5.5% false-positive rate and ACOG recommendations showed the detection rate of 54.6% with 20.4% of false-positive rate^10^.

Because of these limitations, an alternative approach has been suggested, including mean arterial pressure, serum PlGF, and uterine artery pulsatility index by organizations such as the Fetal Medicine Foundation. This alternative approach showed much improved performances, with a detection rate of 70–80% with 10–20% false-positive rate^10,26^. In Asian populations, the AUROC was 0.769 for all preeclampsia and 0.857 for preterm preeclampsia. However, this alternative approach needs an additional measurement for serum biomarker and uterine artery Doppler indices, which is expensive and not achievable in most countries.

Currently, the prediction of pregnancy-associated HTN using routine clinical variables in the first trimester is still an unmet need in clinical practice. In the current study, the proposed model with graph-based SSL using the selected 11 important variables achieved the best average prediction performance (mean AUROC of 0.89 in training set and 0.81 in test set) with higher sensitivity and similar specificity compared to risk factors from clinical guidelines and had a higher AUROC than that of PlGF. This result demonstrates that we can predict pregnancy-associated HTN accurately in early pregnancy through the use of routine clinical variables with the help of graph-based SSL.

### Comparison with previous studies

Recent developments in computational algorithms and machine learning have found significant connections between diverse datasets that cannot be otherwise correlated. Machine learning can be used in prediction or decision model development using real-world data that is too complex to be interpreted by classical statistical analysis. With these advantages, there have been numerous studies for predictive model development using machine learning algorithms in the medical field. In obstetric research, several studies have been performed using machine learning for the prediction of successful vaginal delivery and pregnancy complications such as gestational diabetes, small for gestational age, and other complications^27–31^. A small number of studies have been performed to develop prediction models using machine learning algorithms for the prediction of pregnancy-associated HTN^11–13,32^. Jhee et al. suggested a model that made use of maternal factors and antenatal clinical factors during the early second trimester through 34 weeks^12^ and Sufriyana et al. developed a robust machine learning model using data collected between 24–37 weeks, including uterine artery doppler parameters and biomarkers such as soluble fms-like tyrosine kinase receptor-1 (sFlt-1) and PlGF^11^. Using the nationwide health dataset of Indonesia, Sufriyana also improved prediction power using a random forest algorithm with the inclusion of demographic variables and medical histories starting from early pregnancy and ending at delivery^32^. The Swedish national cohort study may be the first to predict pregnancy-associated HTN using first trimester variables—the model was developed using routinely collected variables at the first parental visit. However, the model failed to show robust performance^13^. Recently, Maric et al. suggested a machine learning model based upon 64 routine clinical variables for early prediction of preeclampsia, with moderate performance (AUORC of 0.79) and sensitivity of 45.2%, and false-positive rate of 8.1%^33^.

To the best of our knowledge, the current study is the first to develop a powerful machine learning model based upon a limited number of routine clinical variables from the first trimester to predict pregnancy-associated HTN. For the development of the best model, we used feature selection methods to choose the most important clinical variables and then applied graph-based SSL. Among various clinical variables retrieved in the first trimester, we selected the most important variables to be incorporated in our prediction model. For this, four different feature selection methods were applied, and geometric mean was calculated to integrate the four different rankings. By this process, we were able to select the top 11 variables including clinical measurements in the pre-gestational and first trimester periods as well as laboratory results in the first trimester. The importance of these selected factors was higher than that of traditional variables, such as the previous history of preeclampsia or presence of medical disease including chronic hypertension, diabetes, renal disease, or autoimmune disease. Indeed, these traditional risk factors in clinical guidelines could detect only 27.3% of pregnancy-associated HTN. The proposed model in the current study could detect the risk of pregnancy-associated hypertension with the sensitivity of 80.2 ± 12.7% and the specificity of 82 ± 11.6% (Table 4).

Among the selected important variables, maternal weight or BMI before pregnancy or in early pregnancy are well-known risk factors for pregnancy-associated HTN^34,35^. Several studies have also reported the importance of BP measurements in early pregnancy for the prediction of pregnancy-associated HTN^36^. Furthermore, abnormal maternal hemoglobin levels in the first trimester have been also reported as risk factors for adverse pregnancy outcomes, including pregnancy-associated HTN^37,38^.

### Strengths

To our knowledge, this study is the first to adopt graph-based SSL to develop a prediction model for pregnancy-associated HTN. Traditional machine learning algorithms such as logistic regression, support vector machine, random forest, and gradient boosting are based on supervised learning, meaning that only labeled data (where patients’ primary outcome is known) are used. However, graph-based SSL performs prediction using the propagation of label information according to the structure of patient graphs derived from their characteristics. Graph-based SSL exploits the knowledge of the input structure from patients while at the same time using the label information provided by labeled data. In the current study, our model from graph-based SSL using both labeled and unlabeled data (${\mathrm{SSL}}_{\mathrm{L}+\mathrm{U}}$) had better performance than our model from graph-based SSL with labeled data only (${\mathrm{SSL}}_{\mathrm{L}}$), as well as models based on other machine learning algorithms. The comparative results shows that our patients’ network with selected variable can capture the similar characteristics between pregnant women with or without pregnancy-associated with HTN. Furthermore, semi-supervised approaches incorporating incomplete follow-up samples can help to increase performance on early prediction of pregnancy-associated with HTN.

Patient data without known clinical outcomes generally cannot be used in analyses, especially when a patient enrolls in a prospective cohort study but is loss to follow-up. In terms of machine learning, patients loss to follow-up become unlabeled data and their information cannot be used in supervised learning approaches. In this study, we used graph-based SSL to predict pregnancy-associated HTN with a large number of unlabeled patients. Graph-based SSL can exploit the underlying structure of input data with graphs (i.e., relationships of all patients in the networks) from both unlabeled and labeled data, using the information from labeled data to inform the role of unlabeled data in prediction^39^.

We also compared the model performance of graph-based SSL for pregnancy-associated HTN prediction to that of PlGF. Mean circulating PlGF levels are lower in patients destined to develop pregnancy-associated HTN, making it a useful biomarker for the phenotype^10,26^. Therefore, PlGF measurement itself serves as a classifier that can predict pregnancy-associated HTN. However, the measurement of PIGF in early pregnancy may not be possible in all pregnant women, especially in low-resource areas. Moreover, measurement of PlGF during early pregnancy in low-risk pregnant women has not been established in routine practice. In order to further demonstrate that the proposed patient network captures a group of patients at risk for pregnancy-associated HTN, we performed an external experiment comparing graph-based SSL against PlGF. For this, we labeled only 6.8% of patients who did not have PlGF measurement, and then compared prediction performances between graph-based SSL and PlGF in the classified remaining samples (93.2% (1,255/1,347)). As a result, our proposed model with graph-based SSL had higher AUROC than that of PlGF.

### Limitations and research implications

Some aspects of this study remain for future work. Although the proposed model using graph-based SSL showed the best performance in the current study, further studies with larger cohorts are needed for external validation. As the current study was conducted in Korean pregnant women with a small number of cases, prospective studies with a larger number of cases are needed with the use of the proposed model to verify its clinical utility in other ethnicities or races. In addition, more studies comparing our model to predictive models using additional markers such as ultrasound indices like uterine artery Doppler measure are needed to not only evaluate performance but also analyze cost-effectiveness. In an algorithmic perspective, the selected clinical variables are used only to construct the patients' network. Then, we present a straightforward implementation of a graph-based algorithm, which is used to propagate and predict the labels through semi-supervised approaches. As a proof-of-concept-study, this study demonstrates that incorporating labeled and unlabeled samples can help to predict early prediction of pregnancy-associated HTN using graph-based SSL. The graph-based SSL with patients’ network have observed all the sample beforehand regardless of presence of outcomes. In other words, there is a disadvantage that the network needs to be re-constructed to predict new patients, but also there is advantage that the prediction performance can be improved if the network is updated with high-quality new samples. In most scenarios, semi-supervised approaches outperform inductive ones in terms of prediction accuracy while they often suffer from high training costs compared to inductive approaches^40^. Clearly, semi-supervised learning is known to increase predictive performance as the labeled data increases, like other supervised learning algorithms (such as artificial neural network and support vector machine)^18,41^. Moreover, in semi-supervised approaches, there are several up-to-date algorithms using network, such as the graph convolutional neural network (GCN)^40,42^. To verify the effectiveness of incorporating all samples regardless of outcomes, we applied graph-based SSL to predict pregnancy-associated HTN using label propagations along with the patient’s network with selected variables. There are many comprehensive graph-based machine learning models and it can be used for classifier of predicting early prediction of pregnancy-associated HTN. The graph neural network could be used for this clinical problem, which can keep track and merge features for each patient when predicting early pregnancy-associated HTN. In addition to the well-known women's health problems such as the prediction of breast cancer prognosis, where relatively large amounts of data are collected and studied, our study shows that the semi-supervised approaches incorporating unlabeled samples is also necessary in the problem of predicting pregnancy complications. Furthermore, excluded samples with missing variables can be incorporated in this analysis after missing value imputations. It can lead to a more sophisticated patient network, and the prediction performance can be improved by increasing labeled samples.

In the current study, we excluded patients with abortion or previable preterm birth, because pregnancy-associated HTN is defined as HTN after 20 weeks of gestation and usually does not present in previable period. As these patients are neither patients with labeled data (normal outcome or pregnancy-associated HTN) nor those with unlabeled data, we excluded them from the initial analysis. However, there is a possibility that exclusion of these patients could result in an error in the development of the prediction model. In addition, we did not have information regarding gestational age at diagnosis of pregnancy-associated HTN. Incorporating gestational age at diagnosis as the prediction outcome in the model would be more helpful in the clinical application of the developed model.

## Conclusion

To solve a common issue to handle missing labels (i.e., patients with incomplete follow-up) in medical scenario, this study presented graph-based SSL to predict pregnancy-associated HTN by incorporating labeled and unlabeled data concurrently. Moreover, patients' networks with selected variables identified the most discriminative features for the problem at hand, which can be even more informative than the one recommended by the clinical guidelines. From the experimental results, we showed that the semi-supervised approach would be more helpful in the clinical application of the developed model. Furthermore, based on graph-based SSL, we could classify patients into high-risk and low-risk groups for pregnancy-associated HTN in early pregnancy with high accuracy. The proposed prediction model with routine variables showed higher sensitivity and similar specificity compared to risk factors from clinical guidelines, and with better predictive performances than PlGF. This result suggests the clinical utility of the proposed model, through the use of routine clinical variables with the help of graph-based SSL.

## Methods

### Data source

This is a secondary analysis of a large prospective cohort study that enrolled healthy pregnant women to examine the risk of pregnancy complications^43–46^. This cohort enrolled singleton pregnant women in early pregnancy (before 14 weeks of gestation) from Incheon Seoul Women’s Hospital and Seoul Metropolitan Government Seoul National University Boramae Medical Center in Seoul, Korea. For this study, we selected consecutive pregnant women in the cohort who were enrolled between October 2014 and October 2019 and followed up until delivery.

This cohort collected clinical, demographic, lifestyle, and health-related variables using a self-reported questionnaire, including factors such as age, parity, medical history, and physical measures before pregnancy (height, weight, body mass index [BMI], and waist circumference). Additional clinical and laboratory data were retrieved directly from patient medical records, including blood pressure [BP], weight, and waist circumference in the late first trimester (10–14 weeks). This study was approved by the Institutional Review Board of Seoul National University Hospital, Seoul Metropolitan Government Seoul National University Boramae Medical Center, and the Public Institutional Review Board designated by Ministry of Health and Welfare, and all experiments were performed in accordance with relevant guidelines and regulations. Patients gave informed consent for the use of clinical information for research purposes.

### Study design

Pregnancy-associated HTN was defined as the occurrence of gestational hypertension, preeclampsia, eclampsia, and/or superimposed preeclampsia. Hypertension was diagnosed as a sustained elevation in BP (systolic BP ≥ 140 mmHg systolic and/or diastolic BP $\ge$ 90 mmHg). Preeclampsia was defined as the presence of both hypertension and features of organ damage such as proteinuria, low platelet, renal insufficiency, liver damage, cerebral symptoms, and pulmonary edema. Eclampsia was defined as convulsion in the setting of preeclampsia^7^. Superimposed preeclampsia was diagnosed as the development of preeclampsia in women with preexisting chronic HTN.

### Conventional risk factors

For comparison with a previously developed prediction model, conventional risk factors recommended by American College of Obstetricians and Gynecologists (ACOG) were evaluated in the study population^19^. According to the guideline, pregnant women were considered to be at high-risk of developing HTN if they have at least one high-risk factor or at least two moderate risk factors. High-risk factors include previous history of preeclampsia, chronic HTN, type 1 or type 2 diabetes, renal disease, multiple gestation, and connective tissue diseases such as systemic lupus erythematosus or antiphospholipid syndrome. The current study includes only singleton pregnancy and therefore multifetal gestation was not evaluated. Moderate risk factors include nulliparity, advanced maternal age (≥ 35 years old), and obesity (BMI > 30 kg/m^2^). All women in the current study were Asian, and therefore ethnicity was not considered. Among moderate risk factors recommended by the ACOG guideline, we did not have information regarding interpregnancy interval, family history of preeclampsia, or history of small-for-gestational age or adverse outcomes in previous pregnancies.

### Measurement of PlGF

Blood samples were taken routinely at 10–14 weeks of gestation, centrifuged, and stored at − 70 °C until measurement of PlGF. PlGF was measured using a commercial enzyme-linked immunosorbent assay (ELISA) kit, in accordance with the instruction of the manufacturer (Quantikine ELISA Human PIGF, R&D systems, USA).

### Statistical analysis

For comparison of clinical variables, continuous data were analyzed using the Student’s *t*-test or Mann–Whitney U test, and categorical data were analyzed using χ^2^ test or Fisher’s exact test, as appropriate. We used an alpha threshold of 0.05 as our significance threshold. All statistical analyses were conducted with R version 4.0.3 (http://www.r-project.org) and MedCalc Statistical Software version 13.3.1 (MedCalc Software bvba, Ostend, Belgium).

### Ranking/selecting variables

To determine important clinical factors for the prediction of pregnancy-associated HTN among the 32 clinical routine variables retrieved in first trimester of pregnancy, we applied four multivariate feature selection methods: logistic regression, support vector machines, random forest with Gini criteria, and random forest with entropy criteria^47–49^. From the result of each feature selection method, we calculated a combined feature ranking defined as ${\left({\prod }_{i=1}^{n}{r}_{i}\right)}^\frac{1}{n}=\sqrt[n]{{r}_{1}{r}_{2}\dots {r}_{n}}$, where ${r}_{i}$ was the order of features in $i$ ^th^ feature selection method. The final selection of important variables was determined through backward elimination, which starts by including all variables and then removes the least important variable one by one when building the prediction model using graph-based SSL.

### Model development and evaluation

The study population was divided into training and chronologically independent test sets based upon year of enrollment (training set enrolled between 2014 and 2017; test set enrolled between 2018 and 2019). Our prediction model was developed using the training set, and the hyper-parameters of each model was tuned and optimized through cross validation. The final model was chosen as the model with the best prediction performance in the training set, and it was evaluated in the chronologically independent test set.

**Figure 4:**
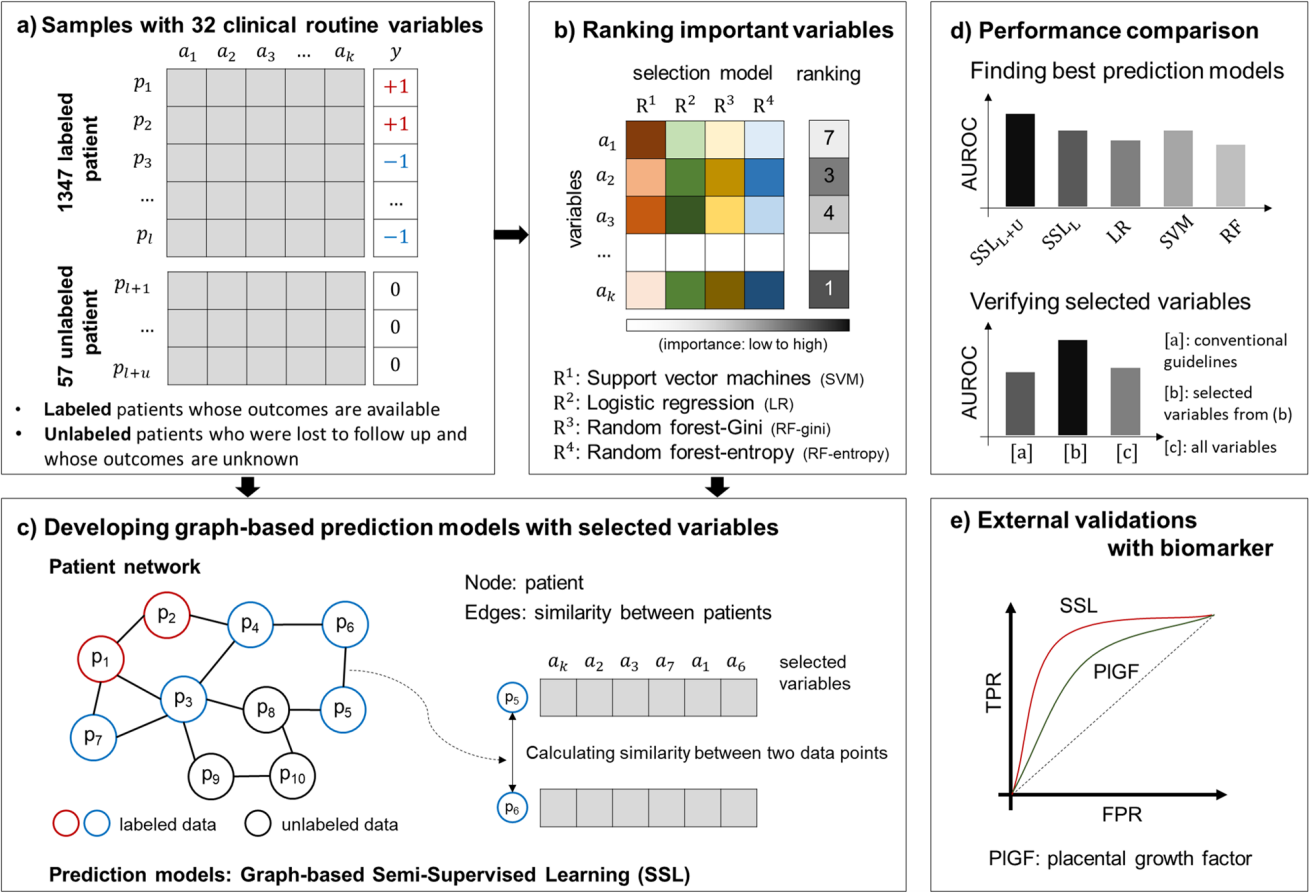
Overall framework.

To develop the prediction model, graph-based SSL was employed to predict pregnancy-associated HTN with constructed patients’ networks (Fig. 4). To demonstrate the generalized prediction performance, we compared five representative predictive models, including graph-based SSL with both labeled and unlabeled data (${\mathrm{SSL}}_{\mathrm{L}+\mathrm{U}}$), SSL only with labeled but without unlabeled data (${\mathrm{SSL}}_{\mathrm{L}}$), logistic regression (LR), support vector machine (SVM)^50^, and random forest (RF)^51^. To investigate the effectiveness of adding unlabeled samples in patients’ network, ${\mathrm{SSL}}_{\mathrm{L}+\mathrm{U}}$ used network with incorporating all labeled and unlabeled samples and ${\mathrm{SSL}}_{\mathrm{L}}$ used network constructed by only 1347 labeled samples. To verify the significance of selected variables, performance comparison was also performed for three different sets of variables: [a] variables from clinical guidelines, [b] selected important variables, and [c] all routine variables. Supplementary Table 2 presents the lists of variables used in set [a], [b], and [c] for the prediction model development. In this manuscript, the type of variable set is marked as the superscript of each model. (e.g., ${\mathrm{SSL}}_{\mathrm{L}+\mathrm{U}}^{[\mathrm{b}]}$ means graph-based SSL with set [b] (selected important variables based on feature selection)). To tune the hyper-parameters and avoid overfitting, fivefold cross validations with stratified random sampling were performed and repeated 100 times in the training set. Note that two transductive models (${\mathrm{SSL}}_{\mathrm{L}+\mathrm{U}}$ and ${\mathrm{SSL}}_{\mathrm{L}}$) were used all training and test set in a network regardless of presence of clinical outcomes, but to compare with the inductive model (SVM, LR, and RF), the experiments for transductive model were conducted by masking the label of the validation set of each fold^14,52^. The area under the receiver operating characteristic curve (AUROC) was used as performance measures.

### Constructing patient network and graph-based semi-supervised learning

For developing the prediction model with graph-based SSL, a patient network was constructed to identify women at high-risk for pregnancy-associated HTN. The network represented the associations between patients. Each node represents an individual patient, and each edge represents the similarity between two patients. The strength of similarity is represented by the weight of the edge—a higher value implies a higher relation between patients.

The patient network is an undirected and weighted graph, $${\varvec{G}}=({\varvec{V}},{\varvec{W}})$$, which is constructed for pregnant women with selected sets of variables. Assume that we have $$n=({n}_{l}+{n}_{u})$$ patients from labeled patients (known outcomes; complete follow-up) $${\varvec{L}}=\{{\left({v}_{i},{y}_{i}\right)}_{i=1}^{{n}_{l}}\}$$ and unlabeled patients (unknown outcomes due to follow-up loss) $${\varvec{U}}=\{{\left({v}_{j}\right)}_{j={n}_{l}+1}^{n}\}$$. Node $${\varvec{V}}=({\varvec{L}}\cup {\varvec{U}})$$ indicates set of patients and similarity $${\varvec{W}}=\left\{{w}_{ij}\right\}$$ indicates edge weights which are calculated between patients using Euclidean distances by $${w}_{ij}=\mathrm{exp}(-\mathrm{dist}({v}_{i},{v}_{j})/{\sigma }^{2})$$. $$\upsigma$$ is hyper-parameter for adjusting distances. We set to the initial label information ($${y}_{l}\in \{\pm 1\}$$ for known outcomes, and $${y}_{u}=\{0\}$$ for unknown outcomes). Here, $${y}_{i}=\{+1\}$$ means that patient $$i$$ was diagnosed with pregnancy-associated HTN, $${y}_{i}=\{-1\}$$ means that patient $$i$$ was not diagnosed, and $${y}_{i}=\{0\}$$ means that the outcomes were unknown since patient $$i$$ does not follow-up. Graph-based SSL is known as a transductive model that can make predictions using both labeled and unlabeled samples, and it showed sufficient prediction performance even if there are many unlabeled samples. Let $${\varvec{y}}={\left({y}_{1},\dots {,y}_{n}\right)}^{\mathrm{T}}$$ denotes the set of labels and $${\varvec{f}}={\left({f}_{1},\dots ,{f}_{n}\right)}^{\mathrm{T}}$$ denotes the set of predicted results. Graph-based SSL perform the predictions on unlabeled samples with the following assumptions: (a) loss condition (predicted value $${f}_{i}$$ should not be close to the given label $${y}_{i})$$, and (b) smoothness condition (predicted value $${f}_{i}$$ should not be different from the $${f}_{j}$$ in adjacent samples). Then, SSL propagates the label information from a patient to the adjacent patient along with the patient network. We can obtain the predicted output $${\varvec{f}}$$ by minimizing the following quadratic objective function as:

$$\underset{f}{\mathrm{min}} {\left({\varvec{f}}-{\varvec{y}}\right)}^{\mathrm{T}}\left({\varvec{f}}-{\varvec{y}}\right)+\upmu {{\varvec{f}}}^{\mathrm{T}}{\varvec{L}}{\varvec{f}}$$

the closed form solution is obtained as ${\varvec{f}}={\left({\varvec{I}}+\upmu {\varvec{L}}\right)}^{-1}{\varvec{y}}$ where the graph Laplacian ${\varvec{L}}$ is defined as ${\varvec{L}}={\varvec{D}}-{\varvec{W}}$, $\mathbf{D}=\mathrm{diag}({d}_{i})$ is degree matrix with ${d}_{i}={\sum }_{j}{w}_{ij}$. The predicted outcome ${\varvec{f}}$ implies whether a likelihood of whether or not the patients have pregnancy-associated HTN. We can decide final labels on each patient by using analysis of the receiver operating characteristic curve. Deciding labels on each patient were performed on ${\varvec{f}}$ as a final predicted outcome with a threshold value. Youden’s $J$ statistics, where the maximum value of $J (=\mathrm{sensitivity}+\mathrm{specificity}-1)$, was used as a threshold value for the receiver operating characteristic curve^53^. The pseudo-code for the proposed method is described in Supplementary Fig. 1.

## Supplementary Information

Supplementary Figure 1.

Supplementary Information.

Supplementary Tables.

## Data Availability

All data generated or analyzed during this study are available on reasonable request from the corresponding author. Implemented code for graph-based semi-supervised learning is provided on https://github.com/dokyoonkimlab/GHTprediciton.
