# Supplementary material for: Development of early prediction model for pregnancy-associated hypertension with graph-based semi-supervised learning
Source: Sci Rep. 2022 Sep 22;12:15793. doi: 10.1038/s41598-022-15391-4 (PMC9499925; doi:10.1038/s41598-022-15391-4)
Supplement: Supplementary file 1 — Supplementary Figure 1. [file 41598_2022_15391_MOESM1_ESM.docx]

**Supplementary Figure 1.** Pseudo-code for predicting pregnancy-associated hypertension

| **INPUT:**   1. **Samples with all routine variables**   samples with known outcomes: $\{\left( v_{i},y_{i} \right)_{i=1}^{n_{l}}\}$, samples with unknown outcomes: $\{\left( v_{j} \right)_{j=n_{l}+1}^{n}\}$  Each samples have k-dimensional feature vectors $\boldsymbol{v}=\{s_{1},s_{2},\ldots, s_{k}\}$   1. **Initialization**   $\mathbf{R}$: order of variables, $\boldsymbol{\Phi}( \cdot)$: any supervised classifier for feature selection (models such as Random Forest)  $\boldsymbol{y}=\left( y_{1},y_{2},\ldots y_{n} \right)^{T}:$ Initial label vector, $\boldsymbol{f}=\left( f_{1},f_{2},\ldots f_{n} \right)^{T}$: prediction output vector   1. **Hyper-parameter**   $\alpha$: Feature selection criteria ($\alpha\geq1)$, $\sigma$: scale-parameter for adjusting distances  $\mu$: trade-off parameter for graph-based semi-supervised learing |
| --- |
| **PROCEDURE**:  **1. Ranking/Selecting variables**  **For** $i$ in 1 to $\vert\boldsymbol{\Phi}( \cdot)$**\|**, where $\left\vert\boldsymbol{\Phi}\left( \cdot\right) \right\vert$ is the number of supervised classifier  $\mathbf{R}_{\mathbf{i}}\mathbf{=}\left\{ r_{i1}\mathbf{,}r_{i2},\ldots\mathbf{,}r_{\mathrm{ik}} \right\}$ $\leftarrow\boldsymbol{\Phi}_{i}\left( \left\{ \left( v_{i},y_{i} \right)_{i=1}^{n_{l}} \right\} \right)$**,** where $r_{i\cdot}$ is order of features obtained from classifier $\boldsymbol{\Phi}_{i}$  **End For**  Combined feature ranking $\hat{\boldsymbol{R}}\leftarrow\left( \prod_{i=1}^{\left\vert\boldsymbol{\Phi}\left( \cdot\right) \right\vert} \boldsymbol{R}_{i} \right)^{1/\left\vert\boldsymbol{\Phi}\left( \cdot\right) \right\vert}$  Selecting $\alpha$-th features as final feature set  **2. Graph-based semi-supervised learning**  Patients’ network $\boldsymbol{G=(V,W)}$**,** where $w_{ij}=exp(-{dist(v_{i},v_{j})}/{\sigma^{2}})$ and $\boldsymbol{v}$ has $\alpha$-dimensional vectors  Run graph-based SSL: $\boldsymbol{f=}\left( \boldsymbol{I+}\mu\boldsymbol{L} \right)^{\boldsymbol{-1}}\boldsymbol{y}$  where graph Laplacian $\boldsymbol{L=D-W}$, and $\mathbf{D}=diag(\sum_{j} w_{ij})$ |
| **OUTPUT:** Predicted results $\boldsymbol{f}$ |
