# Supplementary material for: Development of early prediction model for pregnancy-associated hypertension with graph-based semi-supervised learning
Source: Sci Rep. 2022 Sep 22;12:15793. doi: 10.1038/s41598-022-15391-4 (PMC9499925; doi:10.1038/s41598-022-15391-4)
Supplement: Supplementary file 2 — Supplementary Information. [file 41598_2022_15391_MOESM2_ESM.docx]

**Supplemental Methods**

**Graph-based Semi-Supervised Learning**

The patent network is a undirected and weighted graph, $\boldsymbol{G=(V,W)}$*,* that represents association between patients with nodes and edges. In the network, the set of nodes $\boldsymbol{V}$ denotes a set of patients and the edge weights $\boldsymbol{W}$ denotes associations or relationships between patients. The edge weights $w_{ij}$ between two patients $v_{i}$ and $v_{j}$ are measured by the Gaussian function

$$w_{ij}=\left\{ \begin{matrix} \exp^{-dist \left( v_{i},v_{j} \right)/\sigma^{2}} & if i\sim j \\ 0 & otherwise \end{matrix} \right.$$

where $i\sim j$ indicates that the two patients are connected in network, $dist(\cdot)$ indicates distance measures (in this research, Euclidean distance was used), and $\sigma$ is hyper-parameter for adjusting distances. Since the value of edge represents the similarity between patients, a higher value implies a higher association between patients.

Given the patient network and label information whether patients have an outcome or not, the graph-based semi-supervised learning (SSL) can perform the prediction for pregnancy-associated hypertension (HTN). Assume that we have $n=(n_{l}+n_{u})$ patients from labeled patients $\boldsymbol{L}=\{\left( v_{i},y_{i} \right)_{i=1}^{n_{l}}\}$ and unlabeled patients $\boldsymbol{U}=\{\left( v_{j} \right)_{j=n_{l}+1}^{n}\}$. To initialize label information for graph-based SSL, the labeled patients (known outcomes) were set to $y_{l}\in\{\pm1\}$ while the unlabeled patients (unknown outcomes due to follow-up loss) were set to $y_{u}=\{0\}$. Here, $y_{i}=+1$ means that patient $i$ was diagnosed with pregnancy-associated HTN, $y_{i}=-1$ means that patient $i$ was not diagnosed, and $y_{i}=0$ means that the outcomes were unknown since patient $i$ does not follow-up. Graph-based SSL is known as a transductive model that can make predictions using both labeled and unlabeled samples, and it showed sufficient prediction performance even if there are many unlabeled samples. Let $y=\left( y_{1},\ldots{,y}_{m} \right)^{T}$ denotes the set of labels and $f=\left( f_{1},\ldots,f_{m} \right)^{T}$ denotes the set of predicted results. Graph-based SSL perform the predictions on unlabeled samples with the following assumptions: (a) loss condition (predicted value $f_{i}$ should not be close to the given label $y_{i})$, and (b) smoothness condition (predicted value $f_{i}$ should not be different from the $f_{j}$ in adjacent samples). The predicted output $f$ is obtained by minimizing the following the quadratic objective function as:

$$\min_{f} \left( f-y \right)^{T}\left( f-y \right)+\mu f^{T}Lf$$

Since the objective function is convex, the closed form solution is obtained as follow.

$$f=\left( I+\mu L \right)^{-1}y$$

where the hyper-parameter $\mu$ trades off smoothness and loss condition, $L$ is graph Laplacian defined as $L=D-W$, $D=diag(d_{i})$ is degree matrix and $d_{i}=\sum_{j} w_{ij}$. The predicted outcome $f$ implies whether likelihood of whether or not the patients have pregnancy-associated HTN. We can decide final labels on each patients by using analysis of the receiver operating characteristic curve.

Deciding labels on each patient was performed on $\boldsymbol{f}$ as a final predicted outcome with a threshold value. Youden’s $J$ statistics, where maximum value of $J (=sensitivity+specificity-1)$, was used as a threshold value for the receiver operating characteristic curve.
