# Supplementary material for: Development of early prediction model for pregnancy-associated hypertension with graph-based semi-supervised learning
Source: Sci Rep. 2022 Sep 22;12:15793. doi: 10.1038/s41598-022-15391-4 (PMC9499925; doi:10.1038/s41598-022-15391-4)
Supplement: Supplementary file 3 — Supplementary Tables. [file 41598_2022_15391_MOESM3_ESM.docx]

**Supplementary Table 1. Baseline clinical features of the study population according to completion of follow-up**

|  | **Complete follow-up**  **(n = 1,347)** | **Incomplete follow-up**  **(n = 57)** | ***P-*value** |
| --- | --- | --- | --- |
| Maternal age (years) | 32.3 ± 4.0 | 31.3 ± 3.9 | 0.074 |
| Advanced maternal age (≥35 years) | 397 (29.5%) | 15 (26.3%) | 0.716 |
| Nulliparity | 692 (51.4%) | 37 (64.9%) | 0.062 |
| Pre-pregnancy BMI | 22.3 ± 3.8 | 22.6 ± 4.0 | 0.487 |
| Obesity (BMI > 30 kg/m^2^) | 62 (4.6%) | 3 (5.3%) | 0.745 |
| Previous history of preeclampsia | 12 (0.9%) | 1 (1.8%) | 0.418 |
| High risk according to clinical guidelines | 202 (15.0%) | 9 (15.8%) | 1.000 |

Data are presented as proportion (%) or mean standard ± deviation.

Abbreviations: BMI, body mass index; NICU, neonatal intensive care unit

**Supplementary Table 2. Variables used for prediction model**

| **Variable Sets** | **Description of variables** |
| --- | --- |
| **Set [a]** | **Variables from clinical guidelines:** history of preeclampsia in previous pregnancy, presence of chronic hypertension/ diabetes mellitus/ renal disease/ or autoimmune disease, nulliparity, age, and BMI before pregnancy/ during early pregnancy |
| **Set [b]** | **Selected important variables based on feature selection methods:** Maternal age, previous history of preeclampsia, weight/ BMI before pregnancy, systolic BP/ diastolic BP in early pregnancy, systolic BP/ diastolic BP/ weight/ BMI in late first trimester, and hemoglobin level measured in the first trimester |
| **Set [c]** | **All clinical variables retrieved during routine clinical practice in the first trimester:** history of preeclampsia in previous pregnancy, presence of chronic hypertension/ diabetes mellitus/ renal disease/ or autoimmune disease, nulliparity, age, Height/ weight/ BMI/ waist circumference before pregnancy, systolic BP/ diastolic BP/ weight/ BMI during early pregnancy, systolic BP/ diastolic BP/ weight/ BMI/ waist circumference in the late first trimester, weight gain during first trimester, family history of diabetes, history of gestational diabetes in previous pregnancy, menarche age, presence of medical disease (thyroid disease, liver disease, tuberculosis, or heart disease), and first trimester laboratory results (hemoglobin, hepatitis B serology, syphilis serology, glucosuria) |

Early pregnancy, measured at 7.7 ± 1.2 weeks; late first trimester, measured at 12.4 ± 0.5 weeks

Abbreviations: BMI, body mass index; BP, blood pressure

|  | Models | AUROC | Sensitivity | Specificity | PPV | NPV |
| --- | --- | --- | --- | --- | --- | --- |
| Training set  (Average measurements of 5-fold cross validation) | $\mathbf{SSL}_{\mathbf{L}+\mathbf{U}}^{[\mathbf{a}]}$ | $0.710\pm0.122$ | $0.637\pm0.142$ | $0.646\pm0.169$ | $0.061\pm0.071$ | $0.986\pm0.008$ |
|  | $\mathbf{SSL}_{\mathbf{L}+\mathbf{U}}^{[\mathbf{b}]}$ | $\boldsymbol{0.885\pm0.071}$ | $\mathbf{0.802}\boldsymbol{\pm0.127}$ | $\mathbf{0.820}\boldsymbol{\pm0.116}$ | $\boldsymbol{0.136\pm0.115}$ | $\boldsymbol{0.994\pm0.004}$ |
|  | $\mathbf{SSL}_{\mathbf{L}+\mathbf{U}}^{[\mathbf{c}]}$ | $0.793\pm0.114$ | $0.721\pm0.132$ | $0.746\pm0.154$ | $0.090\pm0.093$ | $0.991\pm0.007$ |
|  | $\mathbf{SSL}_{\mathbf{L}}^{[\mathbf{a}]}$ | $0.735\pm0.113$ | $0.657\pm0.145$ | $0.667\pm0.175$ | $0.065\pm0.059$ | $0.987\pm0.008$ |
|  | $\mathbf{SSL}_{\mathbf{L}}^{[\mathbf{b}]}$ | $0.847\pm0.108$ | $0.778\pm0.127$ | $0.810\pm0.122$ | $0.116\pm0.084$ | $0.994\pm0.005$ |
|  | $\mathbf{SSL}_{\mathbf{L}}^{[\mathbf{c}]}$ | $0.736\pm0.092$ | $0.675\pm0.125$ | $0.687\pm0.129$ | $0.054\pm0.031$ | $0.989\pm0.006$ |
|  | $\mathbf{LR}^{[\mathbf{a}]}$ | $0.783\pm0.113$ | $0.700\pm0.121$ | $0.698\pm0.137$ | $0.061\pm0.044$ | $0.985\pm0.071$ |
|  | $\mathbf{LR}^{[\mathbf{b}]}$ | $0.839\pm0.104$ | $0.776\pm0.114$ | $0.793\pm0.124$ | $0.117\pm0.119$ | $0.994\pm0.005$ |
|  | $\mathbf{LR}^{[\mathbf{c}]}$ | $0.764\pm0.140$ | $0.712\pm0.128$ | $0.736\pm0.156$ | $0.088\pm0.107$ | $0.990\pm0.016$ |
|  | $\mathbf{SVM}^{[\mathbf{a}]}$ | $0.804\pm0.097$ | $0.713\pm0.163$ | $0.742\pm0.158$ | $0.088\pm0.064$ | $0.991\pm0.007$ |
|  | $\mathbf{SVM}^{[\mathbf{b}]}$ | $0.841\pm0.099$ | $0.759\pm0.100$ | $0.782\pm0.114$ | $0.111\pm0.131$ | $0.993\pm0.004$ |
|  | $\mathbf{SVM}^{[\mathbf{c}]}$ | $0.752\pm0.120$ | $0.708\pm0.129$ | $0.710\pm0.168$ | $0.074\pm0.072$ | $0.989\pm0.009$ |
|  | $\mathbf{RF}^{[\mathbf{a}]}$ | $0.675\pm0.124$ | $0.558\pm0.209$ | $0.744\pm0.047$ | $0.047\pm0.028$ | $0.988\pm0.006$ |
|  | $\mathbf{RF}^{[\mathbf{b}]}$ | $0.776\pm0.122$ | $0.702\pm0.159$ | $0.739\pm0.083$ | $0.064\pm0.049$ | $0.991\pm0.005$ |
|  | $\mathbf{RF}^{[\mathbf{c}]}$ | $0.771\pm0.126$ | $0.705\pm0.164$ | $0.731\pm0.097$ | $0.067\pm0.054$ | $0.991\pm0.006$ |
|  | Risk factors | $-$ | $0.412\pm0.230$ | $0.877\pm0.022$ | $0.066\pm0.038$ | $0.986\pm0.005$ |

**Supplementary Table 3. Performance comparison in training set**

Risk factors: conventional risk factors recommended by American College of Obstetricians and Gynecologists

Abbreviations: AUROC, area under the ROC curve; positive predicted value (PPV); negative predicted value (NPV); [a]: models with variables from clinical guidelines, [b] models with selected important variables, and [c] models with all routine variables
